# Role of Melanin in Melanocyte Dysregulation of Reactive Oxygen Species

**DOI:** 10.1155/2013/908797

**Published:** 2013-02-28

**Authors:** Noah C. Jenkins, Douglas Grossman

**Affiliations:** ^1^Oncological Sciences, University of Utah Health Sciences Center, Salt Lake City, UT 84112, USA; ^2^Department of Dermatology, University of Utah Health Sciences Center, Salt Lake City, UT 84112, USA; ^3^Department of Dermatology, Huntsman Cancer Institute, University of Utah Health Sciences Center, Salt Lake City, UT 84112, USA

## Abstract

We have recently reported a potential alternative tumor suppressor function for p16 relating to its capacity to regulate oxidative stress and observed that oxidative dysregulation in p16-depleted cells was most profound in melanocytes, compared to keratinocytes or fibroblasts. Moreover, in the absence of p16 depletion or exogenous oxidative insult, melanocytes exhibited significantly higher basal levels of reactive oxygen species (ROS) than these other epidermal cell types. Given the role of oxidative stress in melanoma development, we speculated that this increased susceptibility of melanocytes to oxidative stress (and greater reliance on p16 for suppression of ROS) may explain why genetic compromise of p16 is more commonly associated with predisposition to melanoma rather than other cancers. Here we show that the presence of melanin accounts for this differential oxidative stress in normal and p16-depleted melanocytes. Thus the presence of melanin in the skin appears to be a double-edged sword: it protects melanocytes as well as neighboring keratinocytes in the skin through its capacity to absorb UV radiation, but its synthesis in melanocytes results in higher levels of intracellular ROS that may increase melanoma susceptibility.

Inactivation or loss of p16^INK4A^ (p16) is a common event in many tumor types although germ-line mutations in *p16* are disproportionately associated with melanoma predisposition [1]. The p16 protein inhibits the kinase activity of cyclin-dependent kinases 4 and 6, preventing the hyperphosphorylation of retinoblastoma-related pocket proteins that are required to release E2F transcription factors necessary for cell-cycle progression. Thus the canonical tumor suppressor function of p16 is to prevent division of stressed or damaged cells by holding them in the late G1-S transition to allow adequate time for DNA repair, or promoting their irreversible exit from the cell-cycle into a senescent state [2]. We have recently reported a potential alternative tumor suppressor function for p16 relating to its capacity to regulate oxidative stress, demonstrating that the depletion of p16 by RNAi in human cells led to increased levels of intracellular reactive oxygen species (ROS) and the oxidative DNA lesion 8-oxoguanine that was independent of cell-cycle phase [3]. We observed that oxidative dysregulation in p16-depleted cells was most profound in melanocytes, compared to keratinocytes or fibroblasts. Moreover, in the absence of p16 depletion or exogenous oxidative insult, melanocytes exhibited significantly higher basal levels of ROS than these other epidermal cell types. Given the role of oxidative stress in melanoma development [4], we speculated that this increased susceptibility of melanocytes to oxidative stress (and greater reliance on p16 for suppression of ROS) may explain why genetic compromise of p16 is more commonly associated with predisposition to melanoma rather than other cancers.

It is not known why melanocytes maintain higher levels of ROS than other cell types, but we hypothesized a role for melanin since its presence is a distinguishing feature of melanocytes and melanin synthesis is known to generate ROS [5]. A previous study found a correlation between levels of melanin and ROS, showing that both were elevated in melanocytes from dysplastic nevi compared to those from normal skin of the same individual [6]. Melanogenesis is pro-oxidative, commencing with the oxidation of L tyrosine to dopaquinone, an enzymatic process that can be inhibited by N phenylthiourea (PTU). To evaluate the role of melanin in melanocyte oxidative dysregulation, we derived melanocytes and fibroblasts from three separate individuals, and cells were cultured in the absence or presence of PTU for 14 days. This was sufficient to deplete most of the melanin in melanocytes (Figure 1(a), left). Intracellular ROS levels were then quantitated by fluorometric analysis following treatment with the cell-permeable fluorophore DCFDA. As previously reported [3], melanocytes exhibited significantly higher ROS levels compared to donor-matched fibroblasts (Figure 1(a), right). By contrast, treatment with PTU resulted in a reduction of basal intracellular ROS levels in melanocytes comparable to those of fibroblasts (Figure 1(a), right). PTU-treated fibroblasts, on the other hand, showed no significant difference in intracellular ROS from their untreated counterparts.

Next we evaluated the pro-oxidative role of melanin in the context of p16 depletion. Donor-matched fibroblasts and melanocytes were transfected with either control or siRNA specific for p16 [3] to deplete endogenous p16 protein (Figure 1(b), lower). Depletion of p16 in both cell types led to increases in intracellular ROS, with ROS levels consistently higher in melanocytes compared to fibroblasts under both control conditions and following p16 knockdown (Figure 1(b), middle). The removal of melanin by PTU (Figure 1(b), upper) was associated with reduction of ROS levels in melanocytes comparable to fibroblasts, even under conditions of p16 depletion (Figure 1(b), middle). These results implicate melanin as the cause of increased oxidative stress in normal and p16-depleted melanocytes.

It is established that chronic oxidative stress and resulting oxidative damage promote carcinogenesis. Melanocytes are more susceptible to oxidative damage due to maintenance of higher levels of ROS [3]. Loss of p16 function through methylation-mediated gene silencing, mutation, or gene deletion, as is commonly found in melanoma [1], would be predicted to further increase ROS levels and correspondingly increase oxidative damage. Elevated levels of ROS in melanocytes are likely compounded by the relative deficiency of this cell type in the repair of oxidative DNA lesions [7]. Both acute and chronic UV radiations induce ROS in the skin, and we have previously shown that the administration of the antioxidant N acetylcysteine prior to and following acute UV exposure delays melanoma onset in a mouse melanoma model [4]. In this same model system, loss of p16 accelerates UV-induced melanoma development [8]. Although melanocytes may be protected by endogenous melanin which can directly absorb UV-generated photons and oxygen radicals [9], at higher UV doses melanin can be oxidized leading to the generation of ROS [10]. However, we have found in the absence of UV exposure that the pro-oxidative nature of melanin production is directly associated with higher melanocyte basal levels of intracellular ROS, which increase significantly following p16 depletion. Thus the presence of melanin in the skin appears to be a double-edged sword: it protects melanocytes as well as neighboring keratinocytes in the skin through its capacity to absorb UV radiation, but its synthesis in melanocytes results in higher levels of intracellular ROS that may increase melanoma susceptibility. Further studies may elucidate whether the pro-oxidative nature of melanin biosynthesis is indeed the basis for predisposition of individuals with inherited p16 mutations that are more likely to develop melanoma over other cancers.

## Figures and Tables

**Figure 1:**
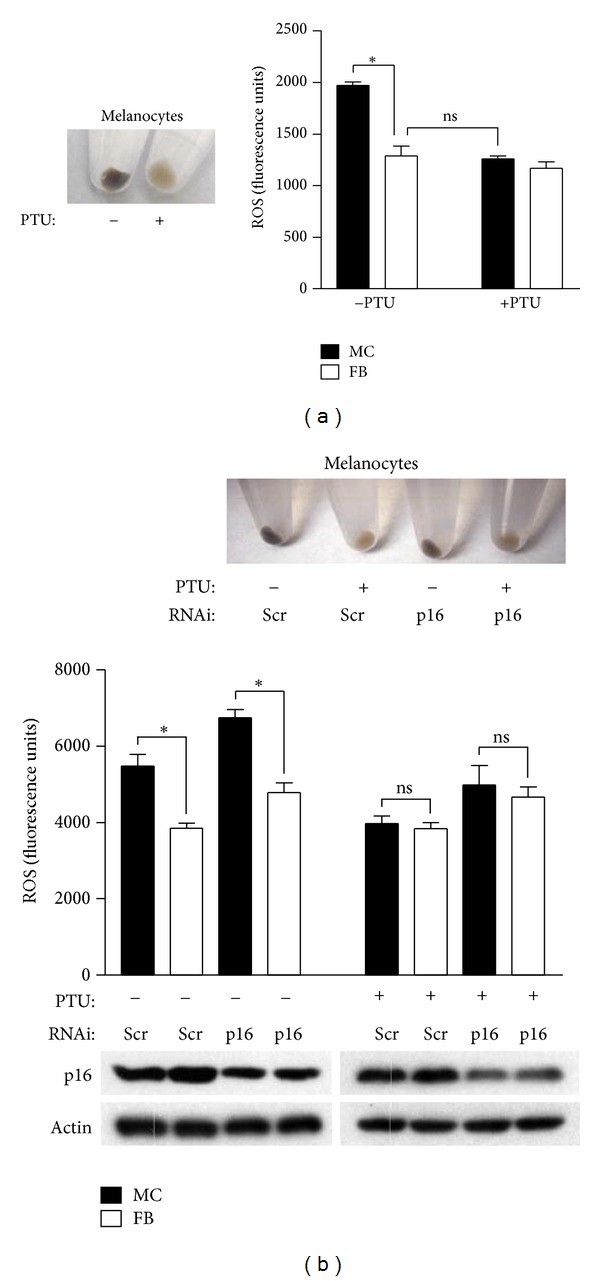
Inhibition of melanin synthesis reduces intracellular ROS in melanocytes. (a) Human melanocytes (MC) were either untreated (−) or treated (+) with 200 *μ*M PTU (Sigma) for 14 days (left panel). Fibroblasts (FB) were isolated from the same donors. Endogenous ROS were detected by the addition of 20 *μ*M DCFDA (Invitrogen) and measured as previously described [3]. Error bars represent S.E.M. of triplicate determinations, and results are representative of two experiments performed. *P = .003 (two-sided *t* test). ns, not significant. (b) PTU treatment of melanocytes transfected with either a control scrambled (Scr) siRNA sequence, or siRNA specific for p16, decreases melanin content (upper panel). Error bars represent S.E.M. of ROS determinations made from three separate donors (middle panel). *P = .04, **P = .03 (paired two-sided *t* test). ns, not significant. Representative Western blot showing p16 levels in siRNAi-transfected cells (lower panel).
